# Supplementary material for: Are carbon emissions trading and green financial instruments synergistic? -Comprehensive quantitative research based on content analysis
Source: PLoS One. 2024 Mar 7;19(3):e0298601. doi: 10.1371/journal.pone.0298601 (PMC10919601; doi:10.1371/journal.pone.0298601)
Supplement: S1 Data — (ZIP) [file pone.0298601.s001.zip › Data/地级市Z_F工作报告.zip!/╕ú╜¿/─■╡┬╩╨/└┤╫╘╣1⁄2╓┌║┼í╛┬φ┐╦╩2╛▌═°í┐_2004.docx]

各位代表：

现在，我代表宁德市人民政府向大会作政府工作报告，请予审议，并请市政协各位委员和其他列席人员提出意见。

一、经济社会发展取得新成效的一年

2003年是全面建设小康社会的开局之年。我市各级政府在省委、省政府和宁德市委的坚强领导下,以“三个代表”重要思想为指导，认真贯彻落实省委、省政府和市委的一系列重大决策部署，坚持把发展作为富民强市的第一要务，团结带领广大干部群众，齐心协力、攻坚克难，抢抓机遇、开拓创新，积极应对非典疫情的挑战，努力克服持续干旱的影响，较好地完成了市一届人大五次会议确定的目标任务。

国民经济较快增长。全年实现国内生产总值271.9亿元，比上年增长10.6%，是1999年以来增幅最高的年份。财政总收入15.5亿元，增长22.7%，其中地方级财政收入9.1亿元，增长18.2%。农业和农村经济稳步发展。在稳定粮食生产的基础上,大力发展多种经营,出台了扶持农业产业化经营、农业特色产业发展和农产品出口等政策措施，有29家企业被列为省、厅两级农业产业化龙头企业。农林牧渔业总产值116.3亿元，增长4.2%。农村社会服务联动网进一步完善。工业经济提速增效。大力推进工业化，加快工业发展，出台了扶持电机电器、船舶修造等产业发展的政策措施，制定了山海协作、优势互补的“飞地”投资政策，全市工业园区引进企业164家。全年工业总产值234.5亿元，增长19.3%，其中规模以上工业产值93.8亿元，增长37.3%，电机电器、船舶修造、医药化工、食品加工、建筑建材、汽摩配件等六类重点产业产值62.3亿元，占规模以上工业产值的66.4%。投资、消费、出口全面增长。全社会固定资产投资74.1亿元，增长4.7%。社会消费品零售总额86.3亿元，增长10.7%。出口总值首次突破2亿美元，增长39.4%。金融运行平稳。年末金融机构各项存款余额164.9亿元，贷款余额143.8亿元，分别增长19.9%和30.4%。

项目带动成效显著。年初确定的38个重点在建项目基本完成年度计划，9个重点项目竣工或基本竣工。尤其是福宁高速公路全线通车，极大地改变了宁德交通滞后状况。洪口电站、穆阳溪梯级闽东电站、闽东医院病房大楼等14个重点项目开工建设。大型临海工业项目引进取得重大突破，投资106亿元的宁德火电厂已开展三通一平。温福铁路、龙安钢铁、汽车改装、宁德城区防洪防潮工程、宁德学院、铁基湾围垦等项目前期工作均取得实质性进展。旅游景区景点开发步伐加快，旅游配套设施建设取得新进展，太姥山被命名为国家地质公园。

体制改革不断深化。进一步理清项目带动、发展县域经济和做大做强中心城市的思路和目标，召开了加快中心城市发展研讨会，着力推进城市经营，重组市国有资产投资营运有限公司、城市建设投资开发有限公司和土地收购储备中心，已取得国家开发银行贷款授信10亿元，为全面开展土地收储，筹集城市建设资金奠定了基础。制定出台支持县域经济发展的简政放权措施，组织开展县域经济“增实力、比后劲”活动，有力推动了县域经济发展。建立民营企业融资担保机制，全市５家融资担保公司帮助中小企业提供担保金额近９亿元。成功实现电力管理体制改革，促进了全市城乡电网体系建设。稳步开展集体林权制度改革，已有20%的村完成林改任务。缓征部分特产税，停征屠宰税，全面推进农村税费改革试点工作，农民直接受益3.4亿元。放手发展非公有制经济，在企业用地、市场准入、对外贸易、技术改造等方面给予扶持。规模以上工业总产值中非公经济产值占69.3%，比上年提高9.1个百分点；民间投资占全社会固定资产投资比重达63.5%，比上年提高12.3个百分点。

对内对外开放力度加大。积极参与区域经济分工与协作，以闽东南、浙南为重点，推进南联北接。大力开展招商引资、项目推介，组织参加第七届中国投资贸易洽谈会、福州国际招商月，成功举办闽东招商节和温州招商推介等活动。宁德与温州政府间开展了交流合作活动，成立了企业家联谊机构，推进两地经济对接，一大批项目在宁德生成落地。全年合同利用外资9380万美元，增长9.9%，实际利用外资7588万美元，增长10.6%。多渠道开拓出口市场，与130多个国家和地区建立贸易伙伴关系。新批外贸进出口经营权企业53家，出口主体多元化的格局开始形成。全年办理出口退税11080万元。积极推动海关、检验检疫等口岸联检部门开展大通关协作，提高了通关效率。新批境外投资生产性企业5家，外向型经济取得新进展。

防非典抗干旱卓有成效。面对突如其来的非典疫情，根据省委、省政府的部署，各级各部门以对人民高度负责的态度，及早部署，沉着应对，采取一系列果断措施，严把输入关，死守福建北大门，保证了人民群众的健康安全，保持了社会稳定、发展加快的良好局面。在抗击历史罕见的严重干旱中，按照“四先”、“四前”的要求，精心组织，周密部署，发动全市上下合力抗旱，科学调度水、电资源，实施人工增雨等措施，基本保证了城乡居民用水、用电，维护了正常的生产生活秩序，使灾害损失减少到最低程度。

人民生活得到改善。认真做好就业和再就业工作，研究出台了“16833”就业工程，全市新增就业岗位2.3万个，帮助7800多名下岗失业人员实现再就业，城镇登记失业率4.3%。“三条保障线”政策得到较好落实，全市有2.7万名城镇企业离退休人员、1万多名失业人员和2.2万名城镇困难群众基本生活得到保障，13.4万名职工纳入基本医疗保险。认真抓好45个省定和28个市定扶贫开发重点村的帮扶工作，多渠道筹集帮扶资金1200多万元，实施帮扶项目190多项。基本完成5000人造福工程和滑坡险情村搬迁任务，硬化农村公路路面400公里，年初确定的15件为民办实事项目基本完成。城乡居民生活进一步改善，城镇居民人均可支配收入和农民人均纯收入分别达到8014元和3246元，增长8.2%和5.2%，恩格尔系数均比上年下降0.2个百分点。

社会事业加快发展。科技创新取得成效，全年实施部、省重点科研项目29项，其中柘荣太子参GAP研究及示范基地建设项目被列入国家科技攻关计划。教育“两基”成果得到巩固提高，小学学龄儿童入学率和初中入学率分别达到99.8％和94.5%。中小学布局调整和危房改造步伐加快，基础教育课程改革稳步实施，素质教育加快推进。高中阶段教育和远程教育加快发展，教育质量有所提高。计划生育工作连续9年完成省里下达的人口控制计划。生态建设和环境保护得到进一步强化。国土资源管理力度加大，土地市场秩序和矿产资源秩序治理整顿工作扎实开展。公共卫生事业加快发展，市、县两级疾病预防控制体系建设得到加强，改造和扩建了部分市、县、乡、村医疗设施。文化事业进一步繁荣，开通宁德电视台公共频道，实现宁德人民广播电台覆盖全市的目标。群众体育、竞技体育不断发展。群众性精神文明创建活动深入开展，城市管理工作得到加强，社区建设取得成效，被评为全省第八届创建文明城市工作先进城市，首次荣获全国“双拥”模范城荣誉称号。民兵预备役工作得到加强，全民国防意识明显提高。人事、编制、审计、民政、民族、宗教、侨务、外事、对台、老区、库区、地震、气象、妇女、儿童、老龄、人防、档案、地方志、新闻出版、残疾人事业和关心下一代等工作都取得新成绩。

民主法制建设扎实推进。自觉接受市人大及其常委会的法律监督和工作监督，办理人大代表建议127件。重视和支持人民政协履行政治协商、参政议政和民主监督的职能，办理政协提案187件。加强同民主党派、工商联、无党派人士和各人民团体的联系，虚心听取各界人士的建议意见。做好村（居）委员会换届选举工作，城镇居民自治、村民自治和村务公开、厂务公开、政务公开等基层民主建设继续加强。依法治市扎实推进，社会治安综合治理不断强化，“严打”整治斗争深入开展，整顿和规范市场经济秩序取得新成效，安全生产整治力度加大，群众信访问题得到高度重视，一些社会不安定因素得到有效化解。政府自身建设取得成效，开展了“创建效能型、服务型、责任型政府，营造优良政务环境”活动，出台了损害经济发展软环境建设行为的责任追究暂行规定，促进了政府工作效率和服务质量的提高。行政审批制度改革继续深化，市政府部门减少行政审批事项和取消行政事业性收费均达到50%以上。认真落实党风廉政建设责任制，反腐倡廉和纠正部门行业不正之风工作取得明显成绩。

各位代表，过去的一年，我们在经受非典和旱情严峻考验下，能够取得这样的成绩，来之不易。这是全市人民团结拼搏、奋力开拓、共谋发展的结果。在此，我谨代表宁德市人民政府，向辛勤工作在各行各业的广大干部群众，向给予政府工作大力支持和有效监督的人大代表、政协委员、各民主党派、工商联、群众团体、无党派人士和离退休老同志，向为宁德改革发展稳定做出贡献的中央及省属驻宁机构、驻宁部队、武警官兵、民兵预备役官兵、公安政法干警，向关心支持宁德建设与发展的港澳台同胞、海外侨胞、国际友人和各界人士，表示衷心的感谢和崇高的敬意！

在肯定成绩的同时，我们也清醒地看到经济社会生活中还存在不少困难和问题。一是三次产业发展不协调，工业化水平较低，经济增长缺乏后劲。二是农业产业化进程比较缓慢，农产品加工水平不高，农民增收难度仍然较大。三是城乡基础设施还不够完善，水利设施比较薄弱，公共卫生体系不健全，教育、科技、文化、卫生事业的发展还相对滞后。四是中心城市经济实力弱，市政设施和城市功能不配套，辐射带动能力不强。五是中心城市和部分县、乡财政困难仍然较大。六是经济社会发展还存在一些体制性、机制性障碍，产权制度改革有待深化，人才不足问题比较突出。七是社会保障、就业再就业压力增大，部分群众生活仍较困难。社会治安、安全生产形势不容乐观。八是一些政府公务人员素质不高，责任心不强，存在作风飘浮、工作不实、方法简单粗暴等现象，影响着软环境的优化和工作质量的提高；个别地方铺张浪费和消极腐败现象依然存在。对此，我们将高度重视，着力加以解决。

二、统筹协调全面推进2004年各项工作

2004年政府工作的总体要求是：以邓小平理论和“三个代表”重要思想为指导，全面贯彻党的十六大、十六届三中全会和中央、全省经济工作会议精神，落实省委、省政府和市委的各项决策部署，坚持以人为本，树立全面、协调、可持续的发展观，以项目带动为抓手，做大做强中心城市，大力发展县域经济，努力改善发展环境，加快南联北接步伐，全面提高对外开放水平，保护好、引导好、发挥好各方面加快发展的积极性。加大产业聚集力度，全力推进工业化、城镇化和农业产业化进程，扩充经济总量，提升经济质量，优化经济结构，不断提高人民生活水平，维护社会安定稳定，促进国民经济持续快速协调健康发展和社会全面进步。

2004年国民经济和社会发展的主要预期目标是：国内生产总值增长10.5%；全社会固定资产投资增长11%；社会消费品零售总额增长10.5%；外贸出口总额增长12%；实际利用外商直接投资增长13%；地方财政收入增长11%；城镇居民人均可支配收入增长7%；农民人均纯收入增长5%；居民消费价格指数增长２%；城镇登记失业率控制在4.5%左右；人口自然增长率控制在7‰以内。

实现上述目标，必须牢牢把握以下几点：

第一，把握加快发展的时代主题。发展是政府的第一要务。贯彻市委“一个目标、三步走、三大战略、四个基地、六项重点”的决策部署，最核心的是做大做强产业，打破三次产业低水平均衡发展格局，突出“工业立市”，以工业化带动城镇化和农业产业化，延伸产业链，实现产业聚集；最重要的是项目带动，扩大对内对外开放，整合和放大各种资源优势，聚集和吸纳各类发展要素，努力化现实优势为现实生产力，变交通提速为经济提速；最关键的是“人才强市”，紧紧抓住稳定、培养、吸引、用好人才四个环节，加强人才资源能力建设，为加快宁德发展提供人才保证和智力支持。

第二，把握统筹发展的基本要求。充分发挥宁德港口、区位、资源优势，按照全面、协调、可持续的科学发展观，坚持以扩充总量为前提，统筹三次产业发展，促进产业互动；以协调发展为中心，统筹区域发展，促进城乡互动；以可持续发展为根本，统筹人口、资源和生态环境保护工作，促进人与自然互动；以人的全面发展为目的，统筹发展各项社会事业，促进经济与社会互动。重点做好“一个中心、两条带”的“三篇文章”：做好“城”的文章，近中期建设滨海港口城市，远期按照“一城、两岛、两区、五港”的空间布局，建设环三都澳海湾城市。以产业为支撑，要素聚集为基础，加快构建以宁德为中心，福安、福鼎、霞浦为依托的沿海城市群，推进城市化进程；做好“港”的文章，大力实施“以港兴市”战略，建设便捷的交通网络，充分凸显港口优势，加快推进临海型、重化型、吞吐型产业发展，增强辐射力，建设沿海经济增长带；做好“山”的文章，加快山区农、林、水、矿产、旅游等资源开发，大力发展资源转换型工业，提升优化传统产业，积极发展特色农业、生态旅游，努力培育具有地方特色和较强市场竞争力的主导产品和产业基地，建设山区经济发展带。

第三，把握优化环境的重要职责。抓环境就是抢抓机遇，抢抓发展。要树立政府营造环境、人民创造财富的理念，把优化环境作为政府工作的主要任务之一。一方面，加快建设一批交通、能源、水利、市政、社会事业项目，形成功能齐全、综合配套的基础设施网络和社会公共服务体系，致力打造经济起飞的硬环境。另一方面，大力加强软环境建设，努力创造公开透明的政务环境、优惠宽松的政策环境、优质高效的服务环境、公平竞争的市场环境、严明规范的法制环境、稳定安全的治安环境和优美舒适的生活环境，努力把宁德建设成为发展的热土、创业的宝地、安居的乐园。

第四，把握改革创新的市场取向。按照市委一届八次全会的部署，加快推进各项市场取向的改革，以思想、体制、机制创新推动各项工作。健全现代市场体系，大力发展商品市场特别是要素市场和各类中介组织，完善市场竞争机制，提高市场监管水平。培育更多更强的市场竞争主体，让市场配置更多社会资源，让企业创造更多社会财富，努力形成微观和宏观都始终充满活力的体制优势。

第五，把握为民谋利的根本宗旨。全心全意为人民服务是人民政府的宗旨,忠实做人民公仆是公务员的天职。必须树立对人民负责的形象，立足于为民求真、为民务实，牢记群众利益无小事，高度重视和认真解决与群众生活息息相关的就业、教育、医疗、社保以及增收减负、社会治安等问题，在排解民忧的基础上凝聚民心，在关心民生的前提下使用民力，在扩大民主的过程中集中民智。每年集中抓好一批经济社会发展方面的重点工作，办好一批与人民群众生产生活密切相关的实事工程，让人民群众从改革开放中看到美好未来，从加快发展中得到更多实惠。

根据上述总体目标和要求，今年重点抓好以下六个方面工作：

（一）扩充总量，优化结构，不断提升经济综合实力

加速推进工业化进程。一是大力发展临海产业。立足区位和港口资源优势，瞄准冶金、石化、能源、重型机械、电子信息、汽车及其配套产业领域，在漳湾、溪南、赛江两岸和沙埕、三沙等临海板块，着力引进一批临海产业项目及其相关配套项目，当前重点抓好宁德火电、龙安钢铁、福安矽钢、船舶修造等大中项目的建设，加快规划发展一批新兴产业集群。二是壮大传统产业规模。立足电机电器、船舶修造、食品加工、建筑建材、医药化工、汽摩配件等传统优势产业，引进和开发上、下游配套项目，延伸产业链，增强传统产业规模效应。重点扶持一批具有核心竞争力的龙头企业，争取新增产值上亿元的企业10家。抓好古田药业公司、霞浦青啤等10个投资千万元以上技改项目。争取新创3个省级以上名牌产品。同时，认真贯彻落实出台的促进中小企业发展的政策措施，设立市、县两级扶持中小企业发展专项基金，重点扶持20家行业骨干企业与50项新增长点项目，促其迅速成长，壮大规模。三是构建工业发展平台。加强工业园区产业布局规划和基础设施配套建设，着力创新园区管理机制，引导工业项目向园区集聚。积极运用市场机制，广泛吸引外资、民资、金融资本参与园区开发。加大闽东工业园区配套建设和招商引资力度，抓好准备动工的鉴明数码科技、汽车改装等一批项目建设。加快县、乡两级工业发展，形成全市上下抓工业、办工业、促工业的良好氛围。

切实加强“三农”工作。一是调整优化农业结构。稳定粮食播种面积，保护粮食综合生产能力，增加粮食储备，确保粮食安全。突出抓好茶叶、水产、畜牧、药材、果蔬、花卉、食用菌等优势农产品基地建设，构建优质高效农产品产业带。加快发展无公害、绿色、有机农产品，争取年内新增绿标产品6个，无公害认证农产品8个。二是大力推进农业产业化。重点扶持全市63家省市级农业产业化龙头企业。争取年内新增2-3家销售上亿元、10家销售上5000万元的农业产业化龙头企业。支持海鸥水产和南阳实业申报国家级农业产业化龙头企业。三是加强农业基础设施和服务体系建设。抓好与省里组织实施水利建设“六千”工程的对接工作，加快建设一批农、林、水重点建设项目，做好河道清水、节水灌溉、山地水利等水利工程建设。完成金涵、溪西2座中型水库和一批小型水库除险加固工程，实施福鼎、福安、古田等三个第四批初级水利化县建设，加快市县两级防汛指挥系统建设，提高农业可持续发展能力。加大农业技术培训和推广力度，巩固完善农村社会服务联动网，鼓励专业化合作，规范养殖业等行业管理，积极培育农副产品购销大户和各种代理商、中介人，提高农产品进入市场的组织化程度。全面落实高致病性禽流感防治工作预案，严防高致病性禽流感传入我市。

不断提升服务业发展水平。一是加快发展现代商贸业和物流业。研究制定城市商业网点规划，推进一批商业中心区、商业街的改造建设，逐步发展连锁经营、电子商务、物流配送等新型流通业态。继续建设一批专业批发市场，抓好10个重点市场的建设改造升级。加快发展会计、审计、律师、资产评估、社会公证、经纪与代理等各类中介服务机构和行业协会、同业商会，提高服务业的整体发展水平。二是大力发展旅游业。尽快完成全市旅游产业发展规划。以沿海高速公路为主轴，加快串点连线，逐步形成以太姥山为龙头的滨海旅游线和以白水洋为重点的山区绿色旅游线。加快白水洋和闽东古廊桥申报世界自然与文化遗产的前期工作，倾力打造白水洋品牌，力争年内完成白水洋主景区旅游公路建设，争取九龙漈-鲤鱼溪和环三都澳两个景区列入国家4A级风景名胜区。继续抓好旅游配套要素建设，增强从业人员素质，改善旅游接待条件。加强与江浙沪、厦漳泉的旅游经济协作，组织好太姥山、白水洋旅游招商活动，促进宁德、温州、福州三市旅游合作区的形成，逐步把旅游业培育成为我市的支柱产业。三是规范发展房地产业。在保持房地产业良好发展势头的基础上，加强宏观调控和政策引导，调整开发结构，提升开发档次和水平，注意调控住宅、商业、写字楼的投放比例，促进房地产市场持续健康发展。

（二）扩大投资，激活消费，保持经济持续快速增长

加大固定资产投资力度。全市安排重点和在建重点项目37个，总投资321亿元，年度投资47亿元；预备重点项目18个，总投资26亿元。要重点抓好“五个一批”项目建设：一是一批交通建设项目。确保福宁高速公路福安连接线、城澳万吨码头年内竣工，开工建设温福铁路，筹建宁德至江西景德镇高速公路，抓好宁屏、宁古、鼎柘公路改造建设，加快城澳万吨级滚装码头、城澳疏港公路、福安湾坞至龙珠疏港公路建设步伐。二是一批电力能源项目。重点抓好宁德火电厂和洪口电站、穆阳溪梯级二、三级电站、寿宁牛头山电站等9个水电项目建设。确保福州至甘棠双回路、甘棠至屏南输变电系统和城区电网改造工程年内竣工，争取开工建设甘棠至福鼎、甘棠至霞浦输变电系统。三是一批市政设施项目。按照市场化运作方式，开工建设宁德、福安、霞浦、古田、柘荣污水处理厂和宁德、福鼎、周宁垃圾处理场，做好铁基湾围垦和东侨东湖治理等项目前期工作。四是一批社会事业项目。抓好闽东医院病房大楼、霞浦一中新校园等项目建设，开工建设东侨实验学校，做好宁德市医院、周宁一中搬迁等项目前期工作。五是一批旅游和市场项目。加快白水洋等一批重点景区景点开发建设，做好三都澳生态农业观光旅游区前期工作，积极申报全国农业生态旅游示范点。加快福安农副产品批发市场、闽东茶叶综合批发市场等一批市场建设，力争闽东农贸中心市场全面竣工投入使用。进一步健全完善重点项目协调、督查工作机制，及时协调解决建设过程中存在的问题，严格落实责任制，抓好工程质量，确保重点项目建设顺利推进。

努力启动民间投资。在城市公用设施和非义务教育、文化、卫生、体育等领域，推出一批项目吸引民间资本进入，努力扩大民间资本在全社会投资中的比重。构筑促进非公有制经济发展的社会化支持服务体系，鼓励和支持非公有制企业进行联合、兼并、收购、重组，做大做强。健全完善中小企业信用担保机构，加快建立和完善市县两级融资担保公司，切实解决非公有制企业融资难问题。依法保护非公有制企业合法权益，表彰一批投资和纳税大户，对贡献大的市外企业家授予“荣誉市民”称号，营造全社会尊重企业家，关心、支持企业发展的良好氛围。

积极扩大消费需求。研究出台鼓励消费的政策措施，引导城乡居民增加即期消费，扩大住房、旅游、教育、汽车、信息、租赁、中介、社会保险等领域的消费，推动新一轮消费升级。加快建立个人信用制度，积极稳妥地发展个人消费信贷，扩大消费信贷范围，加快发展新型消费方式。加大农村市场开拓力度，为农村提供适时、适销及与农村购买力水平相适应的产品。整顿和规范市场经济秩序，建设食品和药品放心工程，严厉打击走私、制假售假、商业欺诈等违法行为，为消费者营造一个放心、公平的市场环境。

切实做好财税金融工作。加大财政对经济的支持力度，努力培植新财源，严格税收征管，努力做到应收尽收。牢固树立过紧日子的思想，调整优化财政支出结构，实行财政预算内外资金统管，努力保稳定、保运转、保改革、保重点。严格财经纪律，做好县乡政府债务清理工作，严禁超财力办事，坚持收支平衡，努力消除财政赤字。深化财政改革，着力构建公共财政框架，完善财政国库管理制度、政府采购制度，不断提高理财水平和财政资金的使用效率，做到生财有方、聚财有道、用财有效。金融工作重点要创新金融业务，开发和拓展金融品种，加强银企协作，促进金融部门在支持地方经济发展中不断壮大，实现双赢。鼓励和支持农村信用社发展，扩大对中小企业的授信额度，试办非全额担保贷款，全面开展小额农户信用贷款和农户联保贷款。完善金融监管体系，防范与化解金融风险。重视保险、证券业的发展。

（三）把握全局，统筹兼顾，扎实推进区域经济发展

着力做大做强中心城市。一是以产业为支撑，带动城市发展。以港口为依托，工业园区为平台，加快项目落地生成，力争有一批项目开工建设和相继投产，以大项目带动主导产业形成。围绕建设沿海发展带，加快培育若干产业集群，重点做好城澳和漳湾港区重型工业项目的规划布局，支持能源项目建设，加快突破钢铁、石化产业的发展。同时，大力发展现代服务业，做好物流基地、物流中心、配送中心的规划建设，加强市场建设，改造传统商业，开发特色旅游，壮大城市经济。二是以规划为龙头，加快城市建设。科学编制中心城市规划，强化规划监管，使规划出形象、出效益、出生产力。坚持以规划指导建设，重点加快实施以东侨开发区路网为重点的一批城市道路工程；以宁德城区防洪防潮工程和第二水源（官昌水库）为重点的一批市政设施工程；以政务中心为重点的一批公共服务工程；以宁德学院、市文化中心艺术馆、体育中心配套场馆为重点的一批公益事业工程。三是以经营为手段，推进城市运营。把土地资本化作为经营城市的核心内容，以土地收储中心为平台，把中心城市规划区内的土地纳入市级统一收储，垄断一级市场，涵养级差地价，实现土地增值。通过开发和回收、置换等方式，盘活现有市直单位资产存量，为政务中心建设筹措资金。推进城市公用设施有偿化经营，使城市建设由简单的生产过程变为资本经营过程。四是以管理为抓手，优化城市环境。进一步明确市、区政府及有关职能部门的职责，加快建立健全市、区两级运转协调的城市管理机制，做到市、区两个积极性充分调动，两种利益统筹考虑。建立适应中心城市发展要求的就业、医疗、教育、社保制度。扎实推进社区建设，完善社区服务功能。加大力度实施“新、绿、亮、洁”工程，加强市民文明素质教育，促进城市文明进步，为2005年实现创建省级“卫生城市”、“文明城市”目标打好基础。

大力发展县域经济。一是加快简政放权。市直有关部门要认真落实省里出台的加快县域经济发展的政策措施，加快职能转变，创新管理办法，把赋予各县（市）经济社会管理权限的政策落到实处，增强县域经济发展活力。各县（市）要把扩权作为加快发展的机遇，用足用活政策空间，不断增强经济综合竞争力。二是坚持分类指导。沿海县（市、区）要挖掘产业特色，立足港口优势，率先承接市外产业、资金、技术转移，大力发展临海产业和海洋经济，更好地发挥辐射带动作用。山区县要立足自身条件，加强外引内联，把当地的土地、劳力、旅游、配套加工等优势，转化为招商引资、城市建设、承接产业转移和发展民营经济的优势，构建各具特色的产业、产品和品牌，促进山区经济发展。进一步抓好县城和省定重点中心镇建设。三是推进山海协作。运用“飞地”投资政策，积极鼓励山区引进项目到沿海落户，鼓励沿海县（市、区）多形式参与山区县项目开发，形成资源共享、优势互补的县域经济发展新格局。做好与省里出台的拓宽山海协作通道、加快欠发达地区发展的政策呼应，继续加大对老、少、边、岛地区支持力度，扶持8个民族乡发展。今年起市对欠发达县在项目、资金、政策上予以倾斜，适当提高欠发达县交通等基础设施项目补助标准，降低其工农业骨干企业进入市级重点的门槛。

（四）深化改革，扩大开放，不断增强经济发展活力

进一步深化体制改革。一是加快产权制度改革。建立归属清晰、权责明确、保护严格、流转顺畅的现代产权制度，完善国有资本合理流动机制，推动资本流动重组。重点抓好市汽车运输公司、九建公司、水电工程局等一批企业的改制工作。以产权制度改革为核心，完成列入全省战略调整实施计划的7家企业改革，加快内贸、外贸、建安、农林水等行业国有企业改革攻坚。二是建立健全国有资产管理体制。推进国有资产管理体制改革，成立市国有资产管理委员会，履行市本级国有资产出资人职能。建立完善有效的激励和约束机制，规范国有资产投资营运有限公司和国有资产授权经营公司运作，确保国有资产保值增值。三是创新投融资体制。落实国务院推进资本市场改革开放和稳定发展的若干意见，加快建立有利于各类企业筹集资金、满足多种投资需求和富有效率的资本市场体系。积极推动安波、万达电机和力捷迅药业等企业尽快进入上市辅导期。组建市产权交易中心，推进各类产权交易、拍卖、转让。积极探索信托投资、股份融资、多方委托贷款等形式，吸引社会资金直接参与重点项目投资开发。四是推进农村各项改革。积极推进农村土地承包经营权流转，促进农业区域化布局和适度规模经营。改革和完善征地制度，积极探索就业安置、社会保险、征地补偿费入股等补偿方式，依法及时对农民足额补偿。继续推进集体林权制度改革，力争今年再完成40%村的集体林改任务。巩固农村税费改革成果，确保农民减负不反弹。五是推动社会领域改革实现新突破。发挥市场机制作用，促进公用事业和公共服务领域形成多元投资、公平竞争、规范经营的发展格局。加快事业单位分类改革步伐，对公益性事业和经营性产业实行不同的管理体制，经营性单位要加快向企业化转制，面向市场，自主经营，壮大实力。公益性事业单位要深化劳动、人事、收入分配和社会保障制度改革，增强活力，改善服务。

进一步提高对内对外开放水平。一是加快南联北接步伐。充分发挥我市区位和资源优势，加快南联福州市和闽东南，北接温州市和浙南地区的步伐，健全政府、企业、民间的多渠道合作机制，主动接受珠江三角洲、长江三角洲等发达地区的经济辐射。二是切实提高招商引资实效。加大招商项目创新力度，盯紧抓实千万美元以上大项目的引进和跟踪落实，在抓好第二产业引资的同时，不断拓宽一、三产业引资新领域，推出一批有深度、带动力强的项目对外招商。在招商形式上外延、内涵并重，通过会展招商、网上招商、以商招商、委托中介招商、产业链招商和登门招商及走出去请进来等方式，形成多元招商新格局。今年要开展面向东南亚国家、华侨华人以及国内宁德商界乡亲的宣传推介和招商工作。积极准备参加世界闽商大会、第八届中国投资贸易洽谈会和第二届中国·福建项目成果交易会，认真筹办闽东招商节、福安电机电器产品订货招商会和闽东旅游节。做好招商项目跟踪落地服务，提高项目履约率和资金到位率，促进招商引资工作上新水平。三是积极推动外贸出口。认真落实鼓励出口的各项措施，调整优化出口产品结构，扩大出口规模。在发展一般贸易的同时，扩大加工贸易、服务贸易和技术贸易，形成多种成份、多种产品共同抢占国际市场的局面。继续加强税贸、关贸、银贸协作，不断提高通关效率。加强与台港澳侨经贸合作，大力推动货物直接往来。四是大力改善外商投资服务环境。要增强人人都是软环境意识，深入开展创建营造优良政务环境活动，认真落实损害软环境建设的责任追究暂行规定，开展行风和政风评议活动，推动机关运行全面提速。进一步完善投资服务机构运作机制，真正做到审批事务的“一站式”、“一条龙”服务。对投资者申办的各种事宜，应特事特办、急事急办。认真解决外资企业生产经营中遇到的困难和问题，重点对近年来外资企业历史积案进行排查，增强外商的投资信心和热情，营造亲商、重商、安商氛围。

（五）关注民生，扩大就业，努力提高人民生活水平

认真做好就业和再就业工作。全面落实促进就业和再就业的扶持政策，继续实施“16833”工程，力争全年新增就业岗位2.5万个，帮助6000名下岗失业人员实现再就业。积极引导农村富余劳动力有序转移，切实做好农民外出务工的组织、指导、培训和服务工作。建立健全各级劳动力转移工作网络，积极筹建劳务派遣公司，抓好屏南、周宁、福鼎、霞浦等4个农村劳动力转移试点县试点工作，力争新增城镇劳动力和农村劳动力转移就业10万名。积极创造依法维权环境，建立预防和制止拖欠农民工工资的长效管理机制，切实维护农民工合法权益。

进一步建立健全社会保障体系。做好企业离退休人员养老保险、失业保险、城市居民最低生活保障“三条保障线”的衔接,抓好企业养老金社会化发放。巩固完善失业保险制度，推进失业保险与最低生活保障制度的衔接。加大解决困难企业职工参加医疗保险的工作力度，完善多层次的医疗保险体系。认真做好《工伤保险条例》的贯彻落实工作，稳步推进工伤、生育保险改革。全面推进农村低保工作，将收入低于保障线的所有农村贫困居民全部纳入低保范围，帮助困难群体提高生活水平。

大力加强扶贫帮困工作。继续抓好45个省级和28个市级扶贫开发重点村的定点扶贫工作。切实解决沿海突出部、海岛、部份山区饮水困难问题，确保年底前完成霞浦三沙引水工程及10个乡镇、200个村供水工程。加快滑坡险情村搬迁和乡村道路改造升级，力争完成800公里农村道路硬化工程建设，完成5000人“造福工程”和滑坡险情村搬迁任务。大力帮助库区移民和上岸定居的连家船民改善生产生活条件。继续做好社会福利、社会救济和慈善事业。

加快公共医疗卫生服务体系建设。继续深化乡镇卫生院体制改革，抓好乡镇卫生院危房改造，扶持“空白点”村卫生所建设，积极开展新型农村合作医疗制度试点工作，进一步健全农村卫生网络。切实加快建设突发公共卫生事件医疗救治体系、疫情信息网络体系、疾病预防控制体系和卫生执法监督体系，重点抓好市疾控中心业务楼、康复医院病房楼和紧急救援中心建设，提高公共卫生突发事件的快速应急反应和处理能力。增强全民防疫意识，确保人民群众身体健康。

（六）以人为本，注重协调，促进三个文明全面发展

深入实施科教兴市和人才强市战略。进一步深化科技体制改革，确立企业技术创新和科技投入的主体地位，认真组织攻关一批省级重点项目，实施一批省级高新工业技术和创新基金项目，大力发展一批民营科技企业。加大对农村教育的投入，抓紧启动第二期农村中小学危房改造工程，稳步推进农村中小学撤点并校，办好农村寄宿制中小学。依法核定义务教育助学金，并通过社会开展扶贫助学活动，解决农村家庭经济困难学生接受义务教育的问题，巩固提高“两基”水平。鼓励社会力量办学，加快高中和职业教育发展步伐，力争年招生计划以8%以上速度增长，为确保到2005年高中阶段教育入学率达60%打好基础。兴办闽东农业职业技术学院。认真解决高校本科以上学历毕业生就业，有针对性地吸引、调配和补充紧缺人才和重点人才。进一步树立尊重劳动、尊重知识、尊重人才、尊重创造的良好风尚，努力做到用事业造就人才，用环境凝聚人才，用机制激励人才，用法制保障人才，用待遇留住人才。

不断拓展可持续发展空间。全面开展创建计划生育优质服务先进县活动，加强流动人口管理，稳定低生育水平，提高出生人口素质。确保今年古田、蕉城、屏南、福鼎、柘荣率先进入计生优质服务省级先进县行列，霞浦、福安力争实现创建目标，寿宁、周宁也要为确保明年实现创建目标打好基础。认真实行耕地保护制度，加强对土地开发利用的管理，推进围垦造地，保持耕地占补平衡。继续强化国家级海域使用管理示范区建设，切实保护好矿产、海洋和水资源，坚持科学合理开发，提高资源使用效益。做好气象工作，完善台风、洪涝、干旱等灾害的预报、预警机制，加强防灾、减灾、救灾工作。加强环境保护，开展环保宣传教育，提高公众环保意识。认真做好高速公路沿线两侧一重山生态环境保护建设工作，抓好环境监测体系建设，切实解决工业污染问题。积极开展霞浦、柘荣两个国家级生态示范县建设，加快水土流失综合治理，努力走出一条生产发展、生活改善、生态良好的发展路子。

大力推进精神文明建设。认真贯彻公民道德建设实施纲要，深入开展“以德治市”教育和建设“诚信宁德”活动。进一步深化文明城市、文明行业、文明村镇创建活动，力争年内分别有三分之一以上的县进入全省、全市创建文明城市先进行列。加强国防教育。抓好民兵、预备役部队建设，扎实做好国防动员工作。深入开展拥军优属和军民共建活动，进一步密切军政军民关系。继续完善城乡文化设施，努力建设一批示范性的标准乡镇文化站，繁荣城乡文化生活。重视民间文化的保护、整理与抢救工作，挖掘闽东畲族文化内涵，弘扬少数民族文化特色。组织好闽东苏区创建70周年纪念活动。切实加强文化市场管理和文物保护工作。做好档案、地方志等各项工作。坚持新闻舆论的正确导向，促进新闻出版、广播电视事业的繁荣。广泛开展全民健身活动，提高竞技体育水平，不断增强人民体质。

切实加强民主法制建设。坚持政府向人大及其常委会报告工作、向人民政协通报情况和政府与人大、政协的联席会议制度，自觉接受市人大及其常委会的法律监督和工作监督，主动接受市政协的民主监督。努力提高人大代表议案、建议和政协委员提案的办理质量。认真听取民主党派、工商联、无党派民主人士和各人民团体的建议和意见，不断完善民主决策程序。加强社区民主建设，健全村民自治，完善各种形式的企事业民主管理制度，进一步推行和规范政务、厂务、村务等公开，创办《宁德市人民政府公报》。认真贯彻党的民族、宗教、侨务政策，发挥工会、共青团、妇联等人民团体的桥梁纽带作用。继续实施“四五”普法规划，深入开展“百千万”基层普法依法治理活动。积极推动法律援助工作，建立健全政府法律顾问制度。依法保护妇女儿童、残疾人和老年人、未成年人的合法权益。进一步落实安全生产责任制和重特大安全事故行政一把手责任追究制，深化安全生产专项整治，及时消除各类安全事故隐患。认真做好群众来信来访和矛盾纠纷排查调处工作，妥善处理人民内部矛盾。以“平安宁德”创建活动为载体，强化社会治安综合治理。严厉打击各种有组织犯罪、暴力犯罪和抢劫、盗窃等多发性侵财犯罪，全力维护社会安定稳定。

三、致力建设人民满意的政府

各位代表，建设一个繁荣富庶的宁德，是全市人民的共同心愿；加快推进富民强市进程，是政府义不容辞的责任。面对新机遇、新形势、新要求，我们必须按照胡锦涛总书记提出的务实、为民、清廉和大力弘扬求真务实精神，大兴求真务实之风的要求，紧抓“第一要务”，牢记“两个务必”，实践“三个代表”，切实加强政府自身建设。

我们一定致力建设忧民所忧、乐民所乐的服务政府。“国以民为本，党以民为基。”要坚持服务为本、群众为本、基层为本，常怀为民之心，多行利民之举，摒弃衙门习气，带着深厚感情进社区、入民居，知民之所想，察民之所虑，亲民之所爱，急民之所需，从人民群众的柴米油盐、衣食住行、安危冷暖等入手，把实事办在群众的心坎上，真正做到情为民所系，权为民所用，利为民所谋。

我们一定致力建设职责明确、敢于负责的责任政府。要适应经济社会转型的需要，对政府的职能和责任进行重新定位，加快转变政府职能，切实把政府的工作重心转到搞好宏观调控、市场监督、社会管理和公共服务上来。要强化责任观念，牢记在其位、谋其政、尽其责，敢于承担责任，勇于挑战困难，善于解决难题。建立健全责任考评机制和责任追究机制，做到矛盾不上交、困难不回避、责任不推诿，真正体现对人民负责，对宁德发展负责。

我们一定致力建设求真务实、运转高效的效能政府。求真务实重在付诸实践，见诸行动，取得成效。要树立科学的发展观和正确的政绩观。要把办实事、抓落实作为政治品质的集中体现，把求实效、创业绩作为能力才干的鉴别标准，以发展论英雄，让想干事的有机会，能干事的有舞台，干成事的有地位。全体政府工作人员都要有务实效的作风、抓落实的办法、敢碰硬的精神、肯吃苦的境界，对基层请示要及时批复，急事急办；对群众的呼声要高度重视，限期解决。要改进会风，提倡少开会、开短会，用更多的精力抓落实。对今年政府工作的各项目标任务，要一件件、一桩桩地落到实处，一抓到底，抓出成效。对各项工作要强化督查，严格奖惩，努力建成人人高效率，事事快节奏的高效政府。

我们一定致力建设说到做到、重信践诺的诚信政府。“人无诚信不立，国无诚信不强。”要围绕提高行政公信力这一核心，开展政府信用建设。坚持讲实话、出实招、办实事、求实效，不急功近利、不好大喜功。做出的决策，要定一条是一条，条条算数；部署的工作，要干一项成一项，项项落实；政府向人代会报告的工作坚持事事追踪，逐项完成；政府发文坚持少而精，做到令行禁止，贯彻到位；政府及组成部门承诺要有度，说了就做，说到做到，说好做好。

我们一定致力建设依法行政、公正严明的法治政府。“法令行则国治，法令弛则国乱。”政府各部门和全体公务员必须切实增强依法办事意识，严格按照法定权限和程序履行职责，把政府各项工作纳入法制化轨道，做到依法决策，依法办事。不断深化行政执法体制改革，强化行政执法监督，完善行政复议制度和过错追究制度，切实规范行政执法行为。做好《行政许可法》宣传实施工作，不断提高依法行政水平。

我们一定致力建设干净干事、清风正气的廉洁政府。“公生明，廉生威"。廉政，就是要求为官者不贪不占，既干事又干净。必须大力发扬清正廉洁、艰苦奋斗的工作作风，坚持厉行节约，勤俭办事，珍惜民资民力，反对奢侈浪费。严格落实党风廉政建设责任制，按照中纪委三次全会提出的“四大纪律八项要求”，全面落实反腐倡廉“六个机制”，继续抓好领导干部廉洁自律、查办违纪违法案件、纠正部门和行业不正之风三项工作。坚持标本兼治，综合治理，惩防并举，注重预防，全面推行领导干部经济责任审计制度，从源头上预防和治理腐败，牢固树立清正廉洁的政府形象。

各位代表，宁德这一方热土，正焕发着前所未有的勃勃生机。时代赋予我们重任，人民寄予我们厚望。我们要全面贯彻落实“三个代表”重要思想和党的十六大精神，按照省委、省政府的决策部署，在市委的领导下，紧紧依靠全市人民，以昂扬的斗志锐意进取，以饱满的热情致力创新，以务实的作风埋头苦干，抢抓机遇，奋发图强，众志成城，谋求跨越，为开创宁德改革开放和现代化建设新局面而努力奋斗！
